# Quality Control Mechanisms in Bacterial Translation

**DOI:** 10.32607/actanaturae.11401

**Published:** 2021

**Authors:** A. S. Zarechenskaia, P. V. Sergiev, I. A. Osterman

**Affiliations:** Lomonosov Moscow State University, Faculty of Bioengineering and Bioinformatics and Belozersky Institute of Physico-Chemical Biology, Moscow, 119992 Russia; Center of Life Sciences, Skolkovo Institute of Science and Technology, Skolkovo, 143028 Russia; Lomonosov Moscow State University, Institute of functional genomics, Moscow, 119992 Russia; Lomonosov Moscow State University, Department of Chemistry, Moscow, 119992 Russia; Sirius University of Science and Technology, Genetics and Life Sciences Research Center, Sochi, 354340 Russia

**Keywords:** translation, bacteria, quality control, termination, trans-translation

## Abstract

Ribosome stalling during translation significantly reduces cell viability, because cells have to spend resources on the synthesis of new ribosomes. Therefore, all bacteria have developed various mechanisms of ribosome rescue. Usually, the release of ribosomes is preceded by hydrolysis of the tRNA–peptide bond, but, in some cases, the ribosome can continue translation thanks to the activity of certain factors. This review describes the mechanisms of ribosome rescue thanks to *trans*-translation and the activity of the ArfA, ArfB, BrfA, ArfT, HflX, and RqcP/H factors, as well as continuation of translation via the action of EF-P, EF-4, and EttA. Despite the ability of some systems to duplicate each other, most of them have their unique functional role, related to the quality control of bacterial translation in certain abnormalities caused by mutations, stress cultivation conditions, or antibiotics.

## INTRODUCTION

**Fig. 1:**
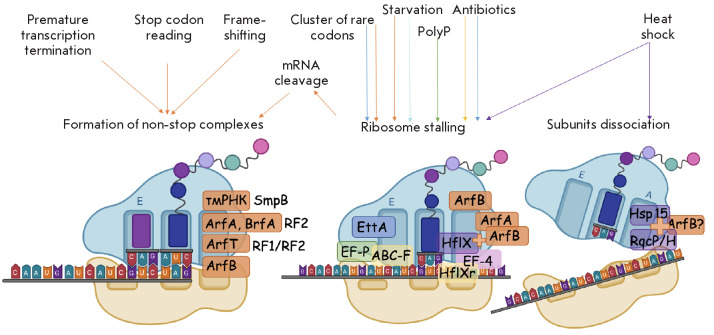
Main causes of translational stalling in a bacterial cell and ways of solving these problems. The figure shows possible causes of translational stalling in a bacterial cell and the tools used by the cell to solve the problems. Left: a non-stop complex formed during translation. This type of substrate is recognized by the factors causing emergency translational termination, followed by the hydrolysis of the peptidyl-tRNA (tmRNA, ArfA, BrfA, ArfB, ArfT). Middle: a ribosome stalled on an intact template. In the case of starvation, this ribosome is stabilized in a hibernation state by Etta; during the passage of a polyproline sequence, EF-P promotes the resumption of translation. Resumption of translation is also provided by EF-4. If this complex is formed under the action of an antibiotic, it can be a substrate for a number of ABC-F proteins, HflX, and, possibly, HflXr. If stalling is caused by a cluster of rare mRNA codons, then the ribosome is likely rescued by ArfB. Right: spontaneous dissociation of ribosomal subunits. The RqcP/H and Hsp15 factors can promote the release of the 50S subunit. (All illustrations are created on BioRender.com)

In a bacterial cell, protein synthesis involves the 70S ribosome that consists of the small 30S and large 50S subunits (*Fig. 1*) [1, 2, 3]. Translation initiation begins with an interaction between the 30S subunit associated with the IF3 factor and the mRNA internal ribosome binding site. Then, the initiation factor IF2 associated with GTP delivers the initiator fMet-tRNA to the P site and IF1 binds to the A site. Initiation is completed by the binding of the 50S subunit, GTP hydrolysis, and the dissociation of initiation factors. During elongation, the ternary complex aa-tRNA (aminoacyl-tRNA)–EF-Tu– GTP binds to the A site of the ribosome. After correct recognition of a codon by the tRNA anticodon, GTP undergoes hydrolysis. The acylated end of the tRNA moves to the peptidyl transferase center (PTC), and EF-Tu is released. Through the transpeptidase reaction catalyzed by the large ribosomal subunit, the peptide chain is transferred to the aminoacyl-tRNA occupying the A site. The EF-G factor catalyzes the movement of the ribosome forward along the mRNA by one codon, after which the deacylated tRNA moves to the E site, and the peptidyl-tRNA enters the P site, thereby freeing the A site for the next aa-tRNA. After dissociation of EF-G, the elongation cycle is repeated. When a stop codon enters the A site, it is recognized by the class I release factors RF1 or RF2, which triggers termination of the protein synthesis. Both factors contain the conserved GGQ motif that catalyzes the hydrolysis of the peptidyl–tRNA bond, thus releasing the newly synthesized peptide. The class II release factor RF3, which also exhibits GTPase activity, promotes the dissociation of RF1 or RF2 from the ribosome. Further, the RRF and EF-G proteins facilitate the disassembly of the 30S and 50S ribosomal subunits and the subsequent binding of IF3 to the small subunit removes the tRNA and mRNA. The translation cycle is complete.

Unlike eukaryotic cells, where translation is preceded by mRNA processing, bacteria are unable to control the quality of the template before protein biosynthesis. Translation in a bacterial cell occurs simultaneously with transcription. This coupling of the two most important processes in time and space, on the one hand, is an advantage: it not only enables the cell to produce proteins at a higher rate, but also underlies the regulatory mechanism of attenuation. On the other hand, the absence of any control over the mRNA before translation inevitably leads to ribosome stalling during the protein synthesis on a template damaged by various factors. The most common cause of these occurrences is ribosome stalling on a damaged mRNA and the formation of the so-called non-stop complex [3]. The list of problems that may arise during translation is not limited only to the lack of a stop codon in the mRNA (*Fig. 1*). Movement of the ribosome can also stop on an intact template; e.g., during translation of “rare” codons and polyproline sequences [4] or under amino acid starvation conditions. Ribosome stalling in the cell also occurs in the presence of antibacterial agents that disrupt protein biosynthesis [5]. Of course, this wide range of potential problems has led to the development of various mechanisms aimed at solving them. In some cases, translation stalling is used to regulate gene expression, so it should not be perceived by the cell as a problem requiring a particular solution [6]. This review discusses the main causes of the problems arising during protein biosynthesis in a bacterial cell and the means used by bacteria to rescue stalled ribosomes. Investigation of some of them is of great practical importance, because the activity of some rescue systems underlies the mechanisms of antibiotic resistance.

The factors that solve the problem of stalled translation may be divided into two types:

1. Factors causing emergency termination of translation, first and foremost, with subsequent hydrolysis of the peptidyl-tRNA and release of the ribosome’ and

2. Factors causing the reactivation of translation in emergency conditions.

Let us consider in more detail the causes behind translation stalling and the rescue systems operating in each specific case.

## FACTORS CAUSING EMERGENCY TRANSLATION TERMINATION WITH SUBSEQUENT PEPTIDYL-tRNA HYDROLYSIS AND RIBOSOME RESCUE

One of the most common problems that the ribosome may encounter during mRNA translation is the absence of a stop codon [3]. This error can occur for a variety of causes. These include premature transcription termination, frameshifting, endo- and exonuclease activity, and stop codon readthrough [3]. Non-stop complexes can also form under the action of some of the endoribonuclease toxins that are necessary for translation arrest under stress conditions [7]. The formation and accumulation of non-stop complexes is toxic to the cell, and the lack of special mechanisms for the elimination of these complexes leads to a rapid decrease in the cell’s ability to synthesize proteins [3, 8, 9]. In this case, the cell viability is affected not only by the deficiency in proteins, the synthesis of which is suddenly interrupted, but also, to a greater extent, by the lack of ribosomes for the translation of other mRNAs. Usually, ribosomes cannot easily dissociate, as they are part of a non-stop complex, since interactions among the peptidyl-tRNA, ribosome, and mRNA firmly hold the complex together [1, 10]. Therefore, bacteria are faced with the primary problem of rescuing stalled ribosomes. Its complexity is related to the need for selective hydrolysis of the desired peptidyl-tRNA. In other words, the mechanism should quite accurately distinguish non-stop complexes from the ribosomes involved in normal elongation.

***Trans*-translation**

The most common mechanism for the rescue of ribosome complexes is the *trans*-translation performed by transport-messenger RNA (tmRNA), which is encoded by the *ssrA *gene, and the SmpB protein. The tmRNA structure and the *trans*-translation mechanism are described in detail in a number of papers [3, 11, 12, 13, 14]. tmRNA derived its name from its ability to combine the functions of both transfer and messenger RNA. The 5’- and 3’-ends of tmRNA form a structure resembling that of Ala-tRNA, which is recognized by alanyl-tRNA synthetase. In addition to a tRNA-like domain, tmRNA contains two to four pseudoknots and a specialized reading frame that encodes a short peptide (8–5 amino acids long, depending on the species). It lacks a start codon, which excludes its normal translation [3].

To perform its function, tmRNA requires the SmpB protein [15]. SmpB stabilizes tmRNA, promotes its recognition by alanyl-tRNA synthetase, and provides binding of the EF-Tu necessary for the delivery of tmRNA to the ribosome. The interaction between tmRNA and EF-Tu is similar to the binding of EF-Tu and aa-tRNA, which is confirmed by the stabilization of this complex on the ribosome in the presence of kirromycin [16].

At the first step of *trans*-translation, the tmRNA– SmpB–EF-Tu–GTP complex binds to the A site of the ribosome. Unlike a ternary complex that interacts with mRNA at the A site, the tmRNA–SmpB–EF-Tu–GTP complex interacts with an empty A site. In this case, the codon–anticodon interaction is replaced by the interaction between SmpB and a ribosome site that binds mRNA on the 3’-side of the P site during normal translation. In this case, the tmRNA–mpB–F-Tu complex triggers GTP hydrolysis. If the mRNA channel is empty, then the tmRNA remains in the A site to continue the translation of the tmRNA coding part. If mRNA is present in the channel, the interaction is prevented because of steric overlap. Thus, the *trans*-translation mechanism does not affect translating ribosomes [17].

**Fig. 2:**
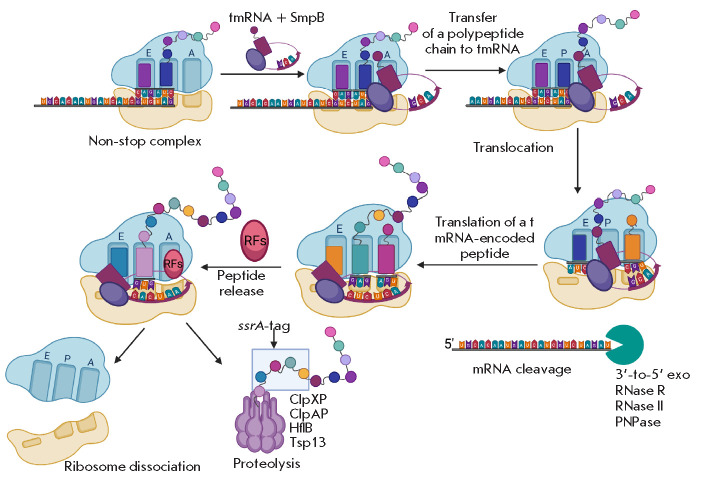
Ribosome rescue by *trans*-translation. The tmRNA–SmpB complex recognizes the ribosome within a non-stop complex and binds in a free A site. Binding of the tmRNA–SmpB complex in the A site leads to the transfer of a polypeptide chain to the Ala-tmRNA and is accompanied by subsequent translocation of the deacylated tRNA from the P site to the E site and the peptidyl-tmRNA–SmpB from the A site to the P site. *Trans*-translation continues until s tmRNA stop codon is reached, which is recognized by the canonical termination factor RF1 or RF2, which stops translation and releases the polypeptide with a tmRNA-encoded tag. Further, the polypeptide is recognized by several proteases, including ClpXP, ClpAP, HflB, and Tsp13, which leads to its rapid degradation [3, 11, 12, 13, 14]

Entry of the tmRNA–SmpB complex into the A site leads to the transfer of a polypeptide chain to Ala-tmRNA and is accompanied by subsequent translocation of deacylated tRNA from the P site to the E site, and the peptidyl-tmRNA–SmpB from the A site to the P site. During translocation, the tmRNA reading frame enters the mRNA channel, such that its first codon, known as the “resume codon,” displaces the C-terminal tail of SmpB from the decoding center.* Trans*-translation continues until a stop codon of the tmRNA is reached, which is recognized by the canonical release factor RF1 or RF2 that terminates translation and releases the polypeptide with a tmRNA-encoded tag. Further, the polypeptide is recognized by several proteases, including ClpXP, ClpAP, HflB, and Tsp13, which leads to its rapid degradation (*Fig. 2*) [3, 18].

The interaction between the protease and the ssrA tag is provided for by the SspB adaptor protein. The original mRNA involved in the non-stop complex is also degraded to avoid repeated translation and a recurrence of emergency situations [3]. In *Escherichia coli* cells, this process is carried out by the RNase R that is recruited by tmRNA–SmpB [19]. Thus, tmRNA plays three important roles in the life of the cell: it is involved in ribosome rescue and in the quality control of the protein and mRNA [13].

**Reserve pathways of ribosome rescue involving ArfA and BrfA**

**Fig. 3:**
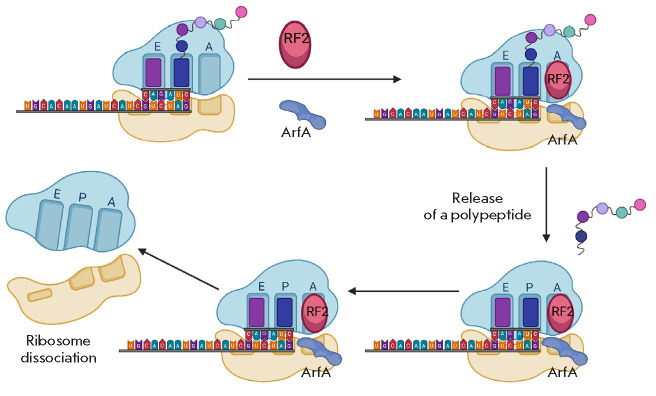
Ribosome rescue by ArfA. ArfA binds at the 3’-end of the mRNA [22] and promotes hydrolysis of the peptidyl-tRNA by RF2

In the case of limited *trans*-translation activity, the ribosome is rescued through an alternative pathway using the Arfa (alternative ribosome rescue factor A) protein. ArfA recruits RF2 to the ribosome, which in turn hydrolyzes the peptidyl-tRNA in non-stop complexes (*Fig. 3*) [20, 21].

ArfA compensates for the absence of a stop codon at the A site and promotes peptidyl-tRNA hydrolysis by RF2 [22]. Therefore, the RF2 GGQ motif hydrolyzing peptidyl-tRNA plays the central role in ribosome rescue by ArfA, while the SPF motif recognizing a stop codon is not that important [23]. In contrast to* trans*-translation, ArfA activity leads only to the release of ribosomes but is not accompanied by a subse quent degradation of nascent polypeptides or mRNA [20, 21, 22, 23, 24]. Interestingly, ArfA recruits only RF2, but not RF1. RF2 is capable of releasing arrested ribosomes with rather low activity, while ArfA enhances this activity [25] through direct interaction with RF2 [26].

should be noted that ArfA is synthesized from non-stop mRNA, and its expression is directly regulated by the *trans*-translation system [27]. In *E. coli*, the ArfA mRNA adopts a hairpin structure and contains an RNase III cleavage site; RNase III removes the stop codon and the final 18 codons of the open reading frame. The *arfA *gene of *Neisseria gonorrhoeae *lacks an RNase III cleavage site; however, the hairpin facilitates transcription termination before the stop codon, thereby providing inhibition of ArfA synthesis [28]. Ribosomes stalled on ArfA mRNA are released during *trans*-translation, and the protein undergoes rapid proteolysis [29]. In some cases, ArfA mRNA can retain a stop codon; then, the classical variant of translation termination with the formation of a fulllength product occurs but the C-terminal region of the full-length ArfA contains a hydrophobic area that promotes protein aggregation, with the protein being cleaved by intracellular proteases. If the activity of* trans*-translation is limited or impaired, then a truncated ArfA lacking the ssrA degradation tag is formed. This truncated product replaces the tmRNA–SmpB system. This regulation mechanism makes ArfA a true reserve ribosome rescue system that operates only when *trans*-translation activity is low or absent [27].

The ribosome rescue mechanism involving the ArfA protein is used by only gram-negative bacteria. In gram-positive bacteria, other mechanisms are present, and, for a long time, the canonical release factors were believed not to be involved in them. However, a mechanism of ribosome rescue similar to the action of ArfA was recently described in *Bacillus subtilis *cells [30]. The protein BrfA (Bacillus ribosome rescue factor A) plays a central role in this mechanism. Like ArfA, it recognizes non-stop complexes and recruits the RF2 release factor to a stalled ribosome. The C-terminal region of the protein also binds to the mRNA channel only if the channel is not occupied by part of the mRNA on the 3’-end of the P site. The similarity with ArfA is also observed at the regulation level: BrfA is synthesized from a non-stop mRNA, and its expression depends on the activity of *trans*-translation. However, the ArfA and BrfA proteins lack structural similarity and are evolutionarily distant from each other. In addition, despite the fact that both proteins recruit RF2, the interaction of each of these proteins with RF2 is different [30]. Probably, gram-positive and gram-negative bacteria developed in parallel reserve ribosome rescue mechanisms to secure the *trans*-translation system.

**ArfB: an alternative rescue system**

An alternative way to rescue stalled ribosomes is provided by the protein ArfB (alternative ribosome rescue factor B). The *arfB *gene was first identified as a lethality suppressor in an *E. coli *mutant lacking both *trans*-translation and the ArfA protein [24]. Homologues of the *arfB *gene were found in 34% of the sequenced genomes of both gram-positive and gram-negative bacteria [31]. Unlike ArfA, ArfB homologues are also present in eukaryotic cells [32].

**Fig. 4:**
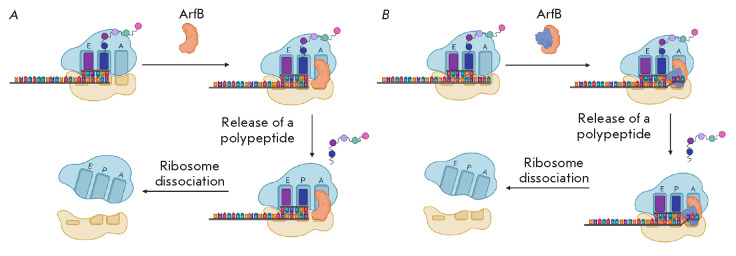
(*A*) – Model of ribosome rescue by ArfB. ArfB binds to the mRNA tunnel of a stalled ribosome. Once bound, the flexible linker region of the protein allows the N-terminal domain to enter the PTC to release a peptide. Then, the ArfB–ribosome complex dissociates [24]. (*B*) – Scenario of ribosome rescue by ArfB when the A site is occupied. If an extended mRNA fragment protrudes from the P site, this fragment moves outside the mRNA tunnel into the intersubunit space and is stabilized there by an additional copy of the ArfB protein [35]. In this case, catalytic ArfB hydrolyzes the peptidyl-tRNA. Then, the ArfB–ribosome complex dissociates

The ArfB N-terminal domain is homologous to the catalytic domains of RF1 and RF2. This domain contains the GGQ motif that plays a crucial role in ArfB-mediated peptidyl-tRNA hydrolysis. In this case, several important amino acid residues necessary for the recognition of the retained complex and binding of the stalled ribosome are located not in the N-, but in the C-terminal domain of the protein. ArfB lacks a domain capable of interacting with a stop codon [33]. Purified ArfB from *E. coli *and *C. crescentus *is able to hydrolyze the peptidyl-tRNA in non-stop complexes *in vitro *in the absence of the RF1 and RF2 release factors (*Fig. 4A*) [24, 31].

The ribosome with a free A site serves as a substrate for tmRNA and ArfA; a similar arrangement was suggested for ArfB, but ArfB was found to interact with ribosomes even when a small mRNA segment extends from the P site [34]. In this situation, the nucleotides of the decoding center are re-arranged, which leads to the expansion of the mRNA tunnel. This plasticity prevents steric overlap of the ArfB C-terminal domain and a short mRNA fragment, thereby facilitating ribosome rescue. The C-terminal domain serves as a sensor that recognizes ribosomes with a free A site or a re-arranged decoding center. After its binding in the mRNA tunnel, a flexible linker region of the protein promotes entry of the N-terminal domain into the PTC to release the peptide. Then, rotation of the ribosome subunits relative to each other leads to the transfer of the deacylated CCA-end of the tRNA to the E site. The ArfB–ribosome complex dissociates, and its subsequent disassembly is facilitated by the ribosome recycling factor RRF [35]. Like ArfA, ArfB releases the ribosome without degradation of a synthesized peptide.

Substrates of ArfB also include ribosomes with a rather extended mRNA fragment (*Fig. 4B*) [35]. In this case, the nucleotides of the decoding center do not change their position and a completely different mechanism operates. The extending mRNA is transferred outside the mRNA tunnel into the intersubunit space and is stabilized there by an additional copy of the ArfB protein, while the catalytic ArfB performs hydrolysis. Therefore, ArfB can act in both monomeric and multimeric forms, which enables the enzyme to efficiently recognize two groups of substrates. Therefore, the protein is able to release stalled ribosomes not only upon template breakage, but also in the case of rare codons or polyproline sequences. This demonstrates the similarity of ArfB to its eukaryotic homologue, the ICT1 protein that, according to some data, releases mitochondrial ribosomes stalled during translation of a cluster of rare codons in [32].

Deletion of *arfB *in *C. crescentus *does not affect viability, but it is lethal in combination with deletion of *ssrA *[31]. However, ArfB cannot fully compensate for the loss of *trans*-translation, because the *ΔssrA C. crescentus *strain has a pronounced growth defect [3]. In addition, unlike ArfA, the synthesis of ArfB is not associated with *trans*-translation activity and it most probably does not act exclusively as a reserve system for *trans*-translation [24, 31]. The action of ArfB, like ArfA, releases the ribosome but does not lead to subsequent targeted degradation of a synthesized peptide or mRNA. Perhaps, ArfB is necessary for the recognition of other possible translation abnormalities: e.g., the release of the ribosome from the non-stop complexes formed due to heat shock [3, 35].

**ArfT releases ribosomes through a different mechanism**

An unusual mechanism of ribosome rescue was found in the causative agent of tularemia, *Francisella tularensis*.* Francisella tularensis *lacks ArfA and ArfB, but inactivation of the ssrA/SmpB system is not a lethal mutation for this bacterium. Transposon mutagenesis followed by deep sequencing revealed a new alternative ribosome rescue factor called ArfT [36].

Deletion of the *arfT *gene was found to lead to a loss of viability only in *F. tularensis *mutants incapable of *trans*-translation. Overexpression of ArfT, on the contrary, promotes the intensive growth of these cells [36]. ArfT is, to some extent, similar to ArfA, and these two factors probably recognize non-stop complexes in a similar way. The C-terminal tail of ArfA binds in an empty mRNA channel of stalled ribosomes using several lysine and arginine residues, including the conserved KGKGS motif. None of these residues by itself is important for the activity of ArfA; however, replacement of individual residues reduces the activity of ribosome rescue *in vitro*. The KKGGSTNKK sequence near the C-terminus of ArfT contains, like ArfA, a number of positively charged residues; therefore, ArfT can probably use this sequence to bind the ribosome [37]. ArfT causes hydrolysis of the peptidyl-tRNA by acting together with termination factors; however, unlike ArfA recruiting only RF2, ArfT interacts with both RF2 and RF1. For example, in the course of *in vitro *modeling of abnormal translation, the addition of ArfT and RF1 from *F. tularensis *to the non-stop complex led to the hydrolysis of the peptidyl-tRNA with an efficiency of 95%, and the addition of ArfT and RF2 from *F. tularensis* led to the same hydrolysis with an efficiency of 84% [36].

Despite the similarity of the C-terminal sequence of ArfT and ArfA, the ability of ArfT to activate both RF1 and RF2 may mean that ArfT interacts with release factors differently than ArfA does. In addition, it is worth noting that ArfT formation is not regulated by translation termination.

## FACTORS CAUSING EMERGENCY TRANSLATION TERMINATION NOT ASSOCIATED WITH PEPTIDYL-tRNA HYDROLYSIS

**HflX**

Heat shock is another cause of translation stalling. In this case, rescue systems interact with the 70S ribosome containing a peptidyl-tRNA in the P site and intact mRNA in the A site. One of the factors that can recognize this substrate is the *E. coli *HflX protein.

**Fig. 5:**
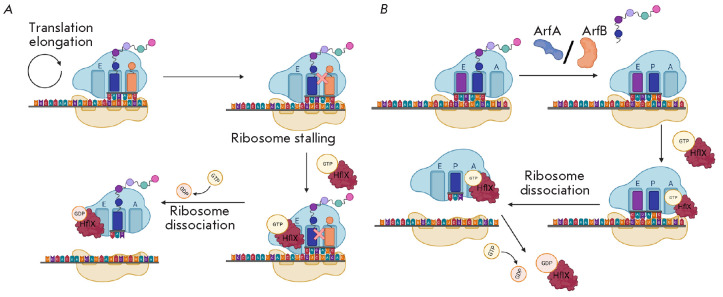
Possible mechanisms of HflX activity. (*A*) – HflX binds to a free E site [38]. The stalled peptide in the PTC is a signal for HflX to hydrolyze GTP. Then, HflX cleaves the 70S subunit into the 50S and 30S ribosomal subunits that can later be used in another round of translation. (*B*) – HflX binds to the A site of a stalled ribosome [39]. The peptide is released by the rescue factor ArfA or ArfB. Then, HflX–GTP binds to the A site and causes dissociation of ribosomal subunits

There are several potential mechanisms of HflX activity. According to one of them, Hf1X can bind to a free E site (*Fig. 5A*) [38]. A peptide stalled in the PTC serves as a signal for the hydrolysis of GTP by HflX. Then, HflX splits the 70S ribosome into 50S and 30S subunits, which can then be used in another round of translation. After splitting of the ribosome, HflX can bind to the A site to prevent re-binding of the 50S and 30S subunits and block binding of other GTPases [38]. HflX was shown to bind to the A site of a stalled ribosome (*Fig. 5B*) [39]. In this model, a peptide is released by the rescue factor ArfA or ArfB. Then, HflX–GTP binds to the A site and causes ribosomal subunits dissociation.

**HflXr**

The action mechanism of numerous antibacterial agents is based on translation suppression. Many of them bind to the PTC, thereby inhibiting the peptidyl transferase reaction [40]. Resistance to these antibiotics is usually associated with the action of efflux pumps or the mechanisms that modify or inactivate an antibiotic molecule [41]. In addition, deletion of the *hflX *gene in the pathogenic bacterium *Mycobacterium abscessus* was recently found to increase sensitivity to macrolide antibacterial agents. The product of this gene is capable of disassembling the ribosomes blocked by macrolides and, as thus, plays an important role in the development of antibiotic resistance in some pathogens [42].

A nontrivial mechanism of resistance, which is probably related to the activity of the HflXr protein, was described in *Listeria monocytogenes *[5, 43]. This protein is a homologue of *E. coli *HflX whose function is to disassemble a stalled ribosome [5]. Although HflXr is also capable of disassembling ribosomal subunits, it cannot be argued that its action is directly related to the displacement of an antibiotic. For example, despite the fact that deletion of the *hflXr *gene renders bacteria more sensitive to erythromycin and lincomycin, the sensitivity phenotype manifests itself only upon simultaneous deletion of another gene, *lmo0919 *[5].

**Release of the ribosome by RqcH and RqcP**

**Fig. 6:**
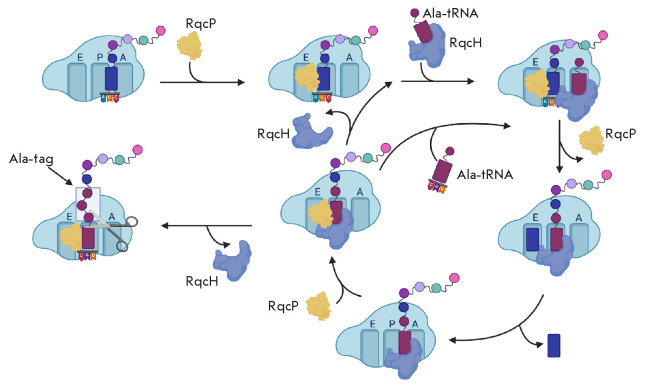
Mechanism of action of the RqcP and RqcH (YabO) proteins. RqcP binds to the 50S subunit and stabilizes tRNA at the P site [44, 45]. RqcH delivers the charged alanine tRNA to the 50S, which occupies a free A site. Further, a polypeptide chain is transferred. Then, RqcP loses its affinity to the ribosome and undergoes a translocation-like movement: in this case, the deacylated tRNA moves to the E site and the peptidyl-tRNA moves to the P site. Later, RqcP rebinds to stabilize the peptidyl-tRNA at the P site. The ribosome-bound RqcH recruits Ala-tRNA. Further, the cycle of this “elongation” can be repeated until the RqcH factor dissociates, and the polypeptide is released. The factor hydrolyzing the peptidyl-tRNA is not exactly known. Probably, it is ArfB

Among the causes behind the abrupt arrest of protein biosynthesis, there is a rather unusual one–premature dissociation of ribosomal subunits. The release of the 50S subunit from a complex with the peptidyl-tRNA occurs using several mechanisms. One of them involves the RqcH and RqcP proteins (*Fig. 6*) [44]. The action of these proteins partially duplicates ssrA/tmRNA activity, because it also produces a polypeptide with a tag recognized by intracellular proteases.

The Rqc2 homolog (RqcH) found in *B. subtilis *is a homologue of the eukaryotic translation quality control factor Rqc2. In a model shown in *Fig. 6*, the RqcP protein binds to the 50S ribosomal subunit and stabilizes tRNA on the P site [44, 45]. RqcH delivers charged alanine tRNA to the 50S, which occupies a free A site. RqcH specifically binds Ala-tRNA due to the fact that the nucleotides G35 and C36 of the tRNA anticodon and the amino acid residues of the RqcH NFACT-N domain form Watson–Crick-like interactions [46]. Further, a polypeptide chain is transferred. Then, RqcP loses its affinity for the ribosome, which facilitates a translocation-like movement of the ribosome: in this case, the deacylated tRNA moves to the E site and the peptidyl-tRNA moves to the P site. Later, to stabilize the peptidyl-tRNA at the P site, RqcP binds again. RqcH either dissociates or, being bound to the ribosome, recruits Ala-tRNA. The cycle of this “elongation” can repeat itself until the RqcH factor dissociates, and the polypeptide is released. The factor that hydrolyzes the peptidyl-tRNA is not clearly known. ArfB is supposed to act in a similar way [44].

**Hsp15**

Actinobacteria and gamma-proteobacteria lack the RqcH and RqcP proteins. However, it should be noted that the RqcP protein is a homologue of the *E. coli* Hsp15 protein [44]. Like RqcH/RqcP, Hsp15 binds to the 50S subunit blocked after sudden disassembly of the ribosome. Hsp15 does not interact with 70S ribosomes, because the small subunit prevents its binding. In the case of unplanned ribosome disassembly, the large subunit becomes accessible to Hsp15. In this case, the peptidyl-tRNA can occupy the A site because of the absence of the 30S subunit. However, this is an unfavorable situation, because the release factor is unable to bind to the 50S subunit in the case of an occupied A site. The Hsp15 protein was found to promote movement of peptidyl-tRNA from the A site to the P site. Then, ArfB presumably performs the release of a polypeptide chain. A significant difference between this mechanism and the action of the RqcH and RqcP proteins is that the synthesized polypeptide chain is not targeted for degradation [47].

**PrfH**

In 1992, the *E. coli *K-12 gene encoding an amino acid sequence with high similarity to the RF1 and RF2 sequences was identified [48]. The element was called PrfH (protein release factor homologue). Later, a significant number of bacterial genomes, even evolutionarily distant from each other, were shown to contain orthologs of this gene. The PrfH protein is similar to the translation termination factors RF1 and RF2 and is regarded as their paralog [49].

There are several suggestions regarding the function of PrfH and which ribosome complex may constitute its substrate. The most plausible hypothesis is that PrfH is a ribosome rescue factor [49].

For example, *prfH *overexpression was found to increase the resistance of *Pseudomonas aeruginosa* bacteria to azithromycin [50]. In addition, by using a reporter system, *prfH *overexpression was shown to decrease the number of stalled ribosome–model mRNA complexes formed in the presence of azithromycin.

However, the role of PrfH is unknown and requires further investigation.

## FACTORS INDUCING TRANSLATION REACTIVATION

**Elongation factor P**

It should be noted that template damage is not the only reason behind ribosome stalling during translation. Ribosomes are often stalled on intact mRNAs. This situation can develop in two scenarios: either elongation resumes, or mRNA is cleaved to form a non-stop complex. Ribosome profiling studies have demonstrated that this ribosome pausing is short-term, because it does not block the movement of other ribosomes translating the same template and does not disrupt gene expression [51, 52]. Many of these cases are caused by elongation delay due to a lack of the necessary aminoacyl-tRNA. In addition, the delay can be caused by pseudoknots and some elements of the mRNA sequence [52].

Stalled ribosomes are capable of spontaneous elongation resumption or translation termination, but specialized translation factors often help in these processes. One of them, EF-P, is a highly conserved protein, a eukaryotic eIF5A homologue, that promotes the synthesis of polyproline sequences [4, 53, 54]. EF-P orthologs in different groups of organisms contain modified amino acid residues whose identity may differ in different taxa [53]. For example, the *E. coli *EF-P contains a lysinyl-hydroxylysine moiety generated by the YfcM [55], YjeK, and YjeA [56, 57, 58] enzymes. EF-P from *P. aeruginosa *contains a rhamnose moiety [59, 60], and the appropriate residue in EF-P from *B. subtilis *is 5-aminopentanol [61]. In eukaryotic cells, eIF5A, the EF-P ortholog, contains a hypusine residue [62].

The formation of a peptide bond between proline residues is complicated and often leads to protein synthesis arrest [63]. Similar difficulties were shown to arise when the ribosome passes three or more consecutive prolines [64]. This motif is found, in particular, in the highly conserved valine-tRNA synthetase [63].

**Fig. 7:**
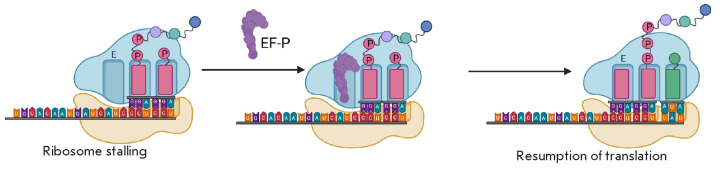
Mechanism of action of the EF-P factor. Binding of EF-P stimulates elongation *in vivo *and *in vitro *when ribosomes are stalled on polyproline sequences. EF-P binds between the E site and the P site on the 50S subunit in close proximity to the peptidyl-tRNA. EF-P is believed to stabilize a PTC substrate conformation productive for the peptidyl transferase reaction [4]

Structural studies of EF-P on the ribosome have shown that EF-P binds between the E site and the P site on the 50S subunit in close proximity to peptidyl- tRNA. Binding of EF-P stimulates elongation *in vivo *and *in vitro *when ribosomes are stalled on polyproline sequences (*Fig. 7*). EF-P is believed to promote the stabilization of the PTC substrate conformation productive for the peptidyl transferase reaction. Despite the fact that EF-P eliminates a small number of abnormalities, it is important enough to the physiology of a bacterial cell. For example, *E. coli *and *S. enterica* strains lacking EF-P have membrane integrity defects and exhibit increased sensitivity to some antibacterial agents [64].

**EF-4 (LepA)**

The well-known conserved translation factor EF-4, also known as LepA, was suggested as a promoter of elongation by catalysis of reverse translocation of stalled ribosomes [3]. However, ribosome profiling data show that EF-4 is involved mainly in the initiation stage and it is not yet known whether this protein plays a role in the rescue of ribosomes [65]. In addition, EF-4 was shown to remodel the A site tRNA, causing a displacement of the tRNA acceptor stem from the PTC. Further research is required to understand the functional significance of A/L distortion of A site tRNA [66].

**EttA**

ATP-binding cassette (ABC) type F proteins that bind to ribosomes and promote dissociation of the ribosome– antibiotic complex are capable of protecting the ribosome against antibiotics [43, 67, 68]. Of particular interest is EttA, an ABC-F protein found in *E. coli *[69]. EttA does not promote antibiotic resistance, but it acts as a translation factor limiting the activity of ribosomes in response to a low ATP level [70, 71]. At high ADP concentrations, EttA binds to the 70S ribosome at the P site, stabilizing it in the so-called hibernation state. This binding interferes with protein synthesis and enables tolerance of adverse conditions by limiting translation

Also, some ABC-F proteins underlie the mechanisms of antibiotic resistance. A detailed review of the ABC-F proteins that protect the ribosome from antibiotics is presented in [40]. These ABC-F proteins bind on the E site of the ribosome. Binding causes a slight counterclockwise rotation of the 30S subunit relative to the 50S, which leads to a shift in the tRNA and allows the ARD domain of the protein to enter the PTC, resulting in a dissociation of the antibiotic. This is presumably associated with the fact that binding of the protein induces allosteric conformational changes in PTC nucleotides containing the antibiotic binding site. The ABC-F proteins found in many bacteria, e.g., *Pseudomonas aeruginosa*, *Staphylococcus aureus*, *Enterococcus faecalis*, and *B. subtilis*, make these organisms resistant to a wide range of antibiotics [40].

## CONCLUSION

The ability to release the ribosomes stalled on mRNA during translation markedly increases the chance of survival and, therefore, has been retained during natural selection (*Table*). Most bacteria need at least one ribosome rescue mechanism to survive. In this case,* trans*-translation has become the most widespread system: the *ssrA *and *smpB *genes are found in more than 99% of bacterial species [3]. Because components of the *trans*-translation system are present in almost all bacterial genomes, and mutations in the genes encoding these proteins reduce cell viability, the proteins involved in this system are considered as attractive targets for new antibacterial agents. These considerations have also been confirmed by the fact that *trans*-translation is specific to bacterial cells, which reduces the risk of possible side effects. Several compounds, potential inhibitors of the release of non-stop complexes through *trans*-translation, have been selected by high-throughput screening [8]. The mechanism of one of them is based on the prevention of polypeptide tagging, while others inhibit proteolysis of tag-containing proteins. One of the compounds inhibits both tag attachment and subsequent proteolysis of the protein.

**Table:** Factors and mechanisms of stalled ribosome rescue

Cause of stalling	Rescue factor	Mechanism of ribosome rescue	Occurrence
Formation of stalled complexes	Trans-translation (tmRNA/SmpB)	Resumption of translation using tmRNA. Tagging of a polypeptide and mRNA	99% of bacterial genomes
	ArfA	RF2 factor recruitment	Gram-negative
	BrfA	RF2 factor recruitment	Bacullus subtilis
	ArfT	Recruitment of RF or RF2	Francisella tularensis
	ArfB	Independent hydrolysis of peptidyl-tRNA	Gram-negative and gram-positive
	HflX	Disassembly of ribosomal subunits	Gram-negative and gram-positive
Abrupt dissociation of subunits	RqcH/RqcP + ArfB (?)	Mimicking of translation elongation for attaching an Ala tag to a polypeptide. Hydrolysis	Except for gamma-proteobacteria and actinobacteria
	Hsp15 +ArfB(?)	Transfer of peptidyl-tRNA to the P-site. Hydrolysis	Gram-negative and gram-positive
Rare codon cluster, polyproline sequence, secondary structure	EF-P	Assistance in peptide bond formation in passing a difficult segment	Gram-negative and gram-positive
	EF-4	Assistance in passing a difficult segment	Gram-negative and gram-positive
	ArfB	Hydrolysis of peptidyl-tRNA	Gram-negative and gram-positive
Action of antibiotics	HflXr	Disassembly of ribosome	Listeria monocytogenes
	ABC-F-proteins	Antibiotic dissociation	Gram-positive
	PrfH-?	Unknown	Gram-negative and gram-positive

The cells of almost all studied bacterial species capable of surviving in the absence of *trans*-translation contain an alternative release factor [72]. For example, the viability of cells with a *ssrA *deletion is maintained by *arfA *in *E. coli*, *brfA *in *B. subtilis*, *arfT *in *F. tularensis*, and *arfB *in *C. crescentus*. *Shigella flexneri *and* N. gonorrhoeae *cannot survive without *trans*-translation [27]. This may be explained by the fact that these pathogens lack an *E. coli *ArfA homologue capable of replacing the tmRNA–SmpB system [27, 73]. Note that ArfT interacts with *F. tularensis *RF1/2 but is unable to bind *E. coli *RF1/2. The BrfA factor interacts exclusively with RF2 of *B. subtilis*. Thus, the described ribosome rescue systems are not interchangeable in different species [26]. In this case, all the alternative rescue systems fail to provide sufficient activity in the absence of *trans*-translation. Deletion of *ssrA *or *smpB* results in many different phenotypes. For example, mutants lacking ssrA may exhibit increased sensitivity to antibiotics and temperature fluctuations and should have virulence defects [27, 74]. *Trans*-translation is preserved in all bacteria, but no species has adapted to the exclusive use of the ArfA, ArfB, or other system. Activity of tmRNA/smpB not only releases stalled ribosomes, but also promotes the removal of nascent polypeptides and damaged mRNAs, which also provides a significant advantage to the system over reserve rescue systems. A partial analogue of *trans*-translation is the RqcH–RqcP system, whose activity leads to the degradation of an incorrect polypeptide.

The additional ribosome rescue systems, both reserve and independent, may hardly be called quality control mechanisms in protein biosynthesis. These systems do not target damaged mRNAs, or the polypeptides synthesized on their basis, for degradation. Despite a variety of reserve mechanisms, none of them duplicates *trans*-translation; there is a suggestion that ribosome rescue is the primary mechanism in translation stalling. Of course, *trans*-translation is the most beneficial of the mechanisms, because it relieves the cell of unwanted and potentially toxic molecules. However, when it is limited or absent, the central need is still implemented – the rescue of stalled ribosomal subunits for subsequent rounds of protein synthesis. Thus, bacteria have acquired a variety of translation rescue systems aimed mainly not at controlling the quality of mRNA but at releasing ribosomal subunits.
